# The Concept of Growth Hormone Deficiency Affecting Clinical Prognosis in IVF

**DOI:** 10.3389/fendo.2019.00650

**Published:** 2019-10-04

**Authors:** John L. Yovich, Sheena L. P. Regan, Syeda Zaidi, Kevin N. Keane

**Affiliations:** ^1^PIVET Medical Centre, Perth, WA, Australia; ^2^School of Pharmacy and Biomedical Sciences, Curtin University, Perth, WA, Australia

**Keywords:** growth hormone GH, growth hormone receptor GHR, follicle stimulating hormone receptor FSHR, bone morphogenetic protein receptor BMPR, luteinising hormone receptor LHR, insulin-like growth factor-I IGF-I, growth hormone deficiency GHD, adult growth hormone deficiency AGHD

## Abstract

The current understanding of human growth hormone (hGH; here GH) action is that the molecule is a 191-amino acid, single-chain polypeptide that is synthesized, stored and secreted by the somatotroph cells within the lateral wings of the anterior pituitary gland. It can be classified as a protein (comprising more than 50 amino acids) but true proteins have tertiary and quaternary chains creating a more complex structure, hence GH is usually classified as a polypeptide. GH is normally secreted at night during sleep and promotes skeletal, visceral and general body growth through the action of somatomedins or IGFs, notably IGF-1. In some tissues, GH action is directed via specific receptors GHRs; these are most abundant in liver, adipose and muscle tissues but have also been shown in granulosa cells, testicular tissues and on the oocyte, as well as in glandular cells of the luteal phase endometrium and decidua; such findings being recent and minimally researched to now. Following engagement with its receptor, the transduction process activates multiple signaling proteins. These all lead to extensive metabolic and mitogenic (growth promoting) responses. Clinically, GH is known to have an important role in pubertal development and is a key hormone for the vigor associated with adolescence and early adult life stages but has a faded presence and role for later adulthood, beyond age 30 years, and is minimally detected in advanced age, beyond 40 years. In association with the rapidly increasing trend for delaying reproduction beyond age 35 years, GH is being widely researched now as a potential adjuvant for infertility treatment in this group who, studies consistently show, have a poorer prognosis than younger females when relying on autologous oocytes. The idea that the age-related reduction in fertility prognosis is a feature of growth hormone deficiency is supported by our studies showing an elevated binding protein IGFBP-3/IGF-1 ratio and this can be reduced to a normal range (matching younger, good prognosis women) by the administration of GH as an adjuvant.

## Introduction

This article explores the physiological processes which might support the clinical findings which indicate a benefit for growth hormone (GH) as an adjuvant in the treatment of women who fail to conceive from *in vitro* fertilization (IVF) treatments. Such women may be categorized as “poor-prognosis” due to a range of categories including poor ovarian responses (POR) to high-dose gonadotrophin stimulation; advanced female age (≥40 years); low ovarian follicle reserve defined by a low antral follicle count (AFC) or low serum level of anti-mullerian hormone (AMH); the failure to generate good-quality embryos after fertilization of their oocytes; or simply the failure to gain successful implantation with resultant pregnancy and livebirth outcomes, so called recurrent implantation failure (RIF). A large number of adjuvants have been explored in the attempt to improve the prognosis for such women, but none of the studies have reached the desired high standard expected from evidence-based medicine (EBM) which requires a number of RCTs to reach meta-analysis support for the particular adjuvant. Where RCTs have been attempted, they have fallen short on recruitment processes and inadequate numbers. This current clinical story has been fully covered in a specific article from our group in this e-book (Yovich et al., under review) which traces the evolution of the “poor-prognosis” concept and indicates that the observational and retrospective studies for GH are strongly supportive of this adjuvant over others which have been reported. Given the recent recognition of the limitations of RCT application in the area of adjuvants or “add-ons” (1), we believe our data reports on GH may be the best achievable currently.

The notion of using growth hormone (GH) as an adjuvant for women in need of assisted reproduction dates back to observations in 1969 followed by studies reported over the 25-year period from 1972 to 1995.

## Historical Studies

One of the earliest to consider the idea was Howard Jacobs, a London-based endocrinologist whose special interest was disorders of ovulation, particularly in association with polycystic ovary syndrome and metabolic disturbance. It was 1969 when Jacobs showed that patients with primary or secondary impairment of adrenal cortical function responded poorly to a wide range of illnesses, injuries or surgical operations, especially those with poorly controlled diabetes (2). This association was determined by measuring 11-hydroxycorticosteroid levels which are very high in appropriate responders but low in those patients who are failing to recover. Concomitant measurements of GH (by a radio-immunoassay sensitive to 0.4 mug/ml) revealed that GH levels are similarly elevated in uncontrolled diabetes, normalizing as insulin response reduces plasma glucose and improves intracellular glucose economy. Jacobs surmised that the hypothalamic-pituitary-adrenal response could somehow also influence GH output in response to various physiological insults, although he was reluctant to draw strong conclusions as GH levels tended to vary widely, even in recovered patients.

Thereafter, the Jacobs' team explored the use of GH in women with amenorrhoea who had shown resistance to ovarian stimulation using human menopausal gonadotrophin (HMG) stimulation (3). The women all had hypopituitarism from a range of causes, many post-surgical from various pituitary tumors, some Kallman's syndrome, others unexplained, and a few with underlying polycystic ovaries. In blinded placebo-RCTs, Jacobs and his team showed that the dosage of HMG required to induce ovulation was significantly reduced (by 30%) and the duration of stimulation was also significantly reduced (by 5 days); when GH was given as an adjuvant. The Insulin-like growth factor-1 (IGF-I) levels rose significantly (by double) but IGF-II levels did not change. These and further findings from Jacobs' team (4–6) “confirmed that GH sensitizes the human ovary to the stimulatory effects of treatment with gonadotrophins.” In a large multi-center study involving several centers from the United Kingdom along with centers in Australia and Sweden, Jacob's group conducted a prospective randomized, placebo-controlled, dose-response study evaluating GH co-treatment with gonadotropins for ovulation induction in hypogonadotropic patients. The findings confirmed the previous studies and showed that GH had an amplification effect of gonadotropin on the ovary and thereby reduced the gonadotropin dosage required to induce ovulation (7). However, whether the effect of GH was exerted directly on the ovary or via the IGF-I system was left unanswered at that time.

Workers from other locations added further useful knowledge. At Stanford University, USA the team of Aaron Hsueh had extensively researched factors influencing growth and organ function, particularly the influence of hormones and growth factors. They also specifically examined the possible direct effect of GH on the differentiation of granulosa cells from the ovaries of hypophysectomised estrogen-treated rats, reporting several studies across the period 1983–1986 (8–10). These *in-vitro* studies revealed that follicle stimulating hormone (FSH) stimulated luteinizing hormone (LH) receptor formation and steroid production in a dose-dependent manner. Concomitant treatment with GH increased LH receptor content by enhancing the action of low doses of FSH. Their data demonstrated that GH augments gonadotropin-stimulated differentiation of ovarian granulosa cells, suggesting an important regulatory role of GH in follicular growth as well as in pubertal development. From similar rat studies in Melbourne, Australia in 1987, the research team of Jock Findlay showed that both GH and IGF-I could independently enhance aromatase activity induced by pregnant mare serum gonadotrophin (PMSG) with elevated estrogen as well as progesterone production; and the stimulatory actions would continue after the gonadotrophin was removed from the culture medium (11, 12). Thus, both GH and IGF-I act on FSH-induced granulosa cells to accelerate the differentiation of the follicular cell to a lutein cell and this was mostly independent of cyclic adenosine monophosphate (cAMP). Findlay's team extended their studies to bovine, sheep, pigs and chicken and showed that a range of growth factors, derived from thecal cells, including epidermal growth factor (EGF) and fibroblast growth factor (FGF) influence not only proliferation but functional differentiation of ovarian follicle cells. Two others, namely transforming growth factor-type ß (TGF-ß) and platelet derived growth factor (PDGF), modulate these actions, sometimes directly opposing them to suggest an inverse relationship between differentiation and mitosis. By 1995, Findlay concluded that there was sufficient evidence supporting the ability of GH to influence ovarian function and proposed that GH was a co-gonadotrophin that synergises with FSH and LH in the promotion of ovarian function. Resolving the unanswered question of Jacobs in 1988, regarding the mechanism (3), he showed this could be manifest in two ways, not necessarily mutually exclusive. On the one hand GH could act via its receptors, resulting in direct modulation of the action of gonadotrophins on ovarian somatic cells. This implied an interaction between the second-messenger systems within the target cell subserving each of the pituitary hormones. On the other hand, GH could act via its receptors to stimulate the production of IGF-I that in turn could have autocrine or paracrine actions on the ovarian somatic cells to modify the actions of FSH and LH. Implicit in this second possibility is the presumption that the ovarian expression of the IGF-I gene and the intra-ovarian actions of IGF-I are either partially or totally GH dependent (13).

Another team from the USA, assembled by Eli Adashi in Maryland, explored growth factor involvement in ovarian maturation with many studies on rat granulosa cells reported across the period 1984–1988 (14, 15). In essence Adashi showed that IGF-I amplified FSH action, consistent with the aforementioned studies. Adashi had in the early 1980's undertaken granulosa cell studies with Hsueh, whom he gratefully acknowledged as one of his mentors (8, 9).

## Clinical Aspects of Growth Hormone Deficiency

Classically, the diagnosis of growth hormone deficiency (GHD) has been confined to pre-pubertal children. The clinical picture has a wide range of manifestations including growth failure, particularly height persistently falling below the fifth centile and children are treated by GH injections through to puberty. Thereafter it had been considered that there is no further need to maintain GH supplementation. However, a number of reports in recent times have shown that adults who had been treated for GHD in childhood had definable conditions in their adult years, particularly related to obesity and diminished cardiac function. For example, a recent Israeli report documenting the post puberty development of 39 persons (23 males and 16 females) with childhood GHD who ceased GH at puberty, exhibited delayed further growth and a progressively increasing development of obesity in their adult years (16). Twelve of them suffered from hyperlipidemia, four developed diabetes mellitus, and five developed serious cardiovascular diseases. One patient died in an accident. None developed cancer. Of the 39 patients, 22 have an education level of high school or higher, and 2 are in special institutions. Most are employed in manual labor. It was concluded that patients with childhood GHD who do not receive early and regular replacement treatment are prone to lag in achieving normal height and suffer from educational and vocational handicaps.

In 2011 The Endocrine Society has issued a report following evaluation of systematic reviews and is now accepting (conceding) the diagnosis of adult growth hormone deficiency (AGHD). That report concludes that “*GHD can persist from childhood or be newly acquired. Confirmation through stimulation testing is usually required unless there is a proven genetic/structural lesion persistent from childhood. GH therapy offers benefits in body composition, exercise capacity, skeletal integrity, and quality of life measures and is most likely to benefit those patients who have more severe GHD. The risks associated with GH treatment are low. GH dosing regimens should be individualized. The final decision to treat adults with GHD requires thoughtful clinical judgment with a careful evaluation of the benefits and risks specific to the individual”* (17).

The Endocrine Society had previously been reluctant to entertain the notion of AGHD as the symptoms are wide-ranging, non-specific and may reflect the natural aging process. The society has been careful not to feed into the idea of widespread use of GH to defer or allay the natural age-related decline in muscle strength and exercise tolerance. Diagnosing newly acquired GHD requires specific testing which can include the insulin tolerance test (ITT) and the growth hormone releasing hormone (GHRH)—arginine stimulation test. These are best undertaken by an endocrinologist as interpretation of the findings can sometimes be complex, although associated low IGF-1 levels tend to clarify the clinical picture. In those adults who had previously been diagnosed as GHD in childhood, low IGF-1 levels alone may be accepted as diagnostic and indicative of the need to re-establish GH therapy. The Endocrine Society also considers the presence of deficiencies in three or more pituitary axes along with low IGF-1 levels, is also sufficient to make the diagnosis without resorting to stimulation testing. This means hypothyroidism, hypogonadism (testosterone or oestradiol deficiency), hypoadrenalism and/or hypo-prolactinaemia combined with low IGF-1 is sufficiently diagnostic to warrant GH therapy.

More recently the NICE Guidelines (18) state that GH therapy for the treatment of adults is recommended only if they fulfill all three of the following criteria:

1. GH deficiency is demonstrated, defined as a peak GH response of <9 mU/l (3 ng/ml) during an ITT or a cross-validated GH threshold in an equivalent test.
2. They have perceived impairment of quality of life (QoL) as demonstrated by a reported score of at least 11 in the disease-specific QoL assessment of AGHD questionnaire.
3. They are already receiving treatment for other pituitary deficiency disorders.

NICE recommends a 3-month period for dosage titration of GH, thereafter a 6-month trial of GH therapy. At 9 months the QoL assessment questionaire should be reviewed with a view to ceasing GH therapy if the score fails to increase by at least seven points. For those who had GH in childhood, the use of GH in adult life is predicated on achieveing peak bone mass, thereafter ceasing unless QoL parameters are reduced.

Furthermore, NICE recommends that the “*Initiation of GH treatment, dose titration and assessment of response during trial periods should be undertaken by a consultant endocrinologist with a special interest in the management of GH disorders. Ongoing treatment should be conducted in a shared-care arrangement with the Endocrinologist as the lead clinician*”.

The above advice should be considered in the context that NICE technology appraisal guidance is about the use of new and existing medicines and treatments in the NHS in England and Wales. In other jurisdictions, the logistics and funding requirements may be quite different leading to compromise in managing AGHD.

Approximately 20–25% of women attending IVF clinics may be categorized as poor-prognosis and be considered for adjuvant therapy, often at the woman's own request. In the consideration of the aforementioned NICE and Endocrine Society guidelines, it may appear that few of these women could fulfill the clinical criteria advised for GH therapy. However, in the context of changing features among the infertile population, we clinicians may need to probe our patients more deeply. Although they may present as ostensibly healthy, the profile of poor responders reflects an older population, prone to higher BMI levels and have subclinical metabolic syndrome (19). If appropriate investigations are performed (with careful cardiovascular assessment, along with lipid profile, GTT and the consideration of ITT where indicated), we may take heed of the first, 1969 citation from Jacobs in this review article; where he demonstrated that reduced GH levels are associated with poor recovery from a range of serious illnesses. Favorable definitive evidence of clinical benefit from GH treatment in AGHD cases is only now beginning to emerge. A recent report involving improvements to specific cardiac prognostic parameters (20) is greeted with cautious optimism. Perhaps women requiring assisted reproduction and are classified as poor prognosis, can be considered to have a subclinical degree of AGHD.

## What Constitutes a Low IGF-1 Level?

Recent reviews concerning GH and IGF-1 show a complex inter-relationship, differing with respect to the natural, pituitary secretion of GH vs. exogenous GH; or hepatic vs. intragonadal (ovary or testis) secretion of IGF-1 (21). The emerging theory is that IGF-1 is an autocrine growth stimulator of follicles and plays a key role at different stages of follicular development. Whilst it appears that IGF-1 is not required for primordial to primary follicle transition, it is necessary for the development of small antral follicles at the gonadotropin-dependent stages (22). Furthermore, IGF-1 increases granulosa cell proliferation, steroidogenesis and oocyte growth (23, 24). It also appears that follicular fluid IGF-1 is a biochemical marker of oocyte quality, providing predictive power of embryo quality and subsequent implantation rates in IVF (25).

Most publications examining IGF-1 ranges report in traditional units (ng/ml) with normal ranges ~180–400 ng/ml but these do have to be adjusted for age. A recent study reporting on healthy Chinese adults shows that gender and age both influence IGF-I levels and there is a gradual decline in levels with advancing age in all adults (26). For example, at age 20 years the median level of IGF-1 is 280 ± 60 ng/ml in males and 300 ± 60 ng/ml in females. At 35 years, the median level is 200 ± 50 ng/ml in males and 220 ± 60 ng/ml in females. At age 45 years, the corresponding levels were 180 ± 40 ng/ml and 200 ± 40 ng/ml, respectively. The matching SI units applies a conversion factor of 76.5 ng/ml equating to 10 nmol/L with a normal range of 200 ng/ml equating to 26.1 nmol/L in males and 20.9 nmol/L for females at the lower standard deviation (SD) point. In practical terms for women aged between 30 and 40 years, the 5th centile (2SDs) equates to 20 nmol/L. However, IGF-1 is known to be carried on six binding proteins, the main one being IGFBP3. In young adults the levels range from 120 to 180 nmol/L and tend to be very stable in individuals. Hence it has been proposed that a ratio may provide the clearest picture regarding IGF bioavailability. This can be reported as IGF-1/IGFBP3 when the ratio for healthy young adults will range 0.15–0.4 (27–30). This can be placed in reverse with IGFBP3/IGF-1 ratio ideally at 3.0 (e.g., 120 nmol/L divided by 40 nmol/L). A ratio <1.6 correlates with an acromegalic state and ratios >4.4 are consistent with GHD. In our own (PIVET, yet unpublished) studies on subfertile women we have regarded IGF-1 levels <20 nmol/L as representing the deficiency range and an IGFBP3/IGF-1 ratio ≥5.0 being consistent with AGHD, implying that such women will be likely to benefit from GH supplementation.

## Why Not Simply Measure GH?

Human GH (hGH, here GH) is a 191-amino acid, single-chain polypeptide hormone that is synthesized, stored, and secreted by the somatotroph cells within the lateral wings of the anterior pituitary gland. Because of its size (comprising more than 50 amino acids), GH could be termed a protein, but it is essentially a linear single-chain polypeptide without the complex foldings with tertiary and quaternary chains which typify true proteins. GH action is directed via specific receptors GHRs; these are most abundant in liver, adipose and muscle tissues but have also been shown in granulosa cells, testicular tissues and on the oocyte, as well as in glandular cells of the luteal phase endometrium and decidua; such findings being recent and minimally researched to now (20). The transduction process for GH is via the Janus kinase signal transduction and activation of transcription (JAK-STAT) signaling pathway after induction of GHR dimers which then activate two JAK2 molecules. This, in turn results in phosphorylation on multiple GHR tyrosines, in turn activating multiple signaling proteins including STATs 5A and 5B, insulin receptor substrate IRS, phosphoinositide PI-3 kinase, extracellular signal-related kinase ERK or mitogen activated protein kinase MAPK. The serine/threonine specific kinase B (PKB, also designated Akt) is also involved in the resulting protein synthesis and inhibition of apoptotic processes. These all lead to extensive metabolic and mitogenic (growth promoting) responses. Pituitary-derived GH, the main serum source, is normally secreted in a 90-min pulsatile fashion, mostly at night during sleep and activates cell-surface receptors directly. However, locally produced GH is continuously generated and activates receptors on the endoplasmic reticulum. These somatotrophins both promote skeletal, visceral and general body growth through the action of somatomedins or insulin-like growth factors although the pattern varies. In particular GH raises serum IGF-1 and IGF-II levels and these proteins are also known as Somatomedin C and A, respectively. They are both growth-promoting proteins with IGF-II mainly active during fetal gestation and IGF-I during adult life. Both somatotrophin (GH) and somatomedins (IGF's) have a variety of effects on lipid, protein and carbohydrate metabolism. The somatomedins stimulate somatostatin from the hypothalamus which suppresses GH release and this creates a negative feedback mechanism on both GH as well as on their own production. Although the liver is the main source of circulating IGF's, the somatomedins are also produced within many tissues where they have both autocrine and paracrine actions in addition to their endocrine action. The current immunoassays have improved the methodology over previous radio-immunoassays, but small peptide interference continues to affect their reliability. So too, does the pulsatile nature of GH release create extremely wide variability in GH detection, such that zero levels can still be consistent with clinical normality. On the other hand, the IGF-I assays have improved with very low coefficients of variation over a wide range lending clinical consistency and reliability (26).

Clinically, GH is also known to have an important role in pubertal development and is a key hormone for the vigor associated with adolescence and early adult life stages for both males and females, but has a faded presence and role for later adulthood, beyond age 30 years, and is minimally detected in advanced age, beyond 40 years. This pattern coincides with the current challenge of managing infertility where female age is the overwhelming limiting factor and future strategies include oocyte preservation at young age, strategies to improve oocyte quality *in-vitro* and stem cell transformations (31). So far, the idea of GH therapy as a specific treatment for older subfertile women has yet to be suggested, let alone studied in an appropriately designed research trial.

## IGF-I Levels in Assisted Reproduction

So far there are few reports covering the diagnosis of AGHD in assisted reproduction, and those being only case reports or observational on small case numbers (32). At PIVET, we have included IGF-1 and IGFBP3 testing on all new consultations over the past 5-years as part of their formal Assessment Cycle. Furthermore, cases provided with GH as adjuvant treatment on the basis of poor prognosis categorization have their levels and ratios reviewed 4-weeks after commencement of GH. Such data will be presented as a retrospective analysis. In the meantime, we have some earlier pilot data from 190 women attending for IVF (presented, but not published) which encouraged our current studies. These showed that, across all age categories (<35, 35–39, ≥40 years), IGF-I levels ranged from 9 to 52 nmol/L with a mean of 24.4 nmol/L. Whilst the younger women (<35 years had a higher mean level (25.7 nmol/L) than women ≥35 years (23.1 nmol/L) the ranges were equally wide. The IGFBP3 levels ranged 101–237 nmol/L with an overall mean of 162.5 nmol/L and tending to be higher in the women ≥35 years. Calculation of the IGFBP3/IGF-l ratios showed levels ranging from 3.3 to 13.8 with a mean of 7.2 (well above our cut-off limit of 5.0; implying AGHD affecting the majority of infertility cases. The mean of 6.7 for women <35 years was less marked than the ratio of 7.6 for those ≥35 years. Of greater interest was the finding of a marked improvement in IGF-I levels for 20 women treated with GH, rising from a mean level of 20 up to 34 nmol/L and which corresponded with a reduction of ratios from a mean high of 8.9 to a mean normal of 4.1. Although this data has not yet been tested by publication, it provides support for our continuing studies on GH as an adjuvant in IVF. In this respect it was reported by the Jacobs team in 1995 (7) that IGF-1 levels rose according to the dosage of GH applied. In their placebo-controlled study GH was administered by intramuscular injection alternate days over the course of gonadotropins to a maximum 7 injections (total dosage ranging 28 IU to 144 IU; as the higher dosages were not required beyond five or six injections). The 4 IU GH dosage caused an incremental rise of IGF-I by a mean of 10 nmol/l; 12 IU GH caused a rise of 20 nmol/L and 24 IU caused IGF-1 levels to rise by a mean of almost 30 nmol/L. Jacobs concluding remark in his 1995 report is pertinent stating “although the actual therapeutic role of GH in ovulation induction is at present unclear, the reality of its interaction with gonadotropins in now unequivocally established.”

## Receptor Studies Involving GH, FSH, LH, and BMP

The first report of GH receptor (GHR) expression in the ovaries came from Israel in 2008 following studies on terminated fetuses as well as from girls and women requiring gynecological procedures (33). The proteins and mRNA transcripts for GH and GHR were detected in oocytes, granulosa cells and stroma cells from both sources (fetuses and women/girls), albeit with low staining intensity only in a portion of the fetal granulosa cells. This supported the earlier studies of GH involvement in ovarian function.

Co-author Sheena Regan has focused her studies on hormonal receptors in the ovary studying both sheep (the highly fecund Booroola sheep which carries a BMP mutation) (34) and human (focusing on women classified as poor-prognosis) (35–37). These human studies demonstrated dysregulation of the granulosa cell density of BMP 1B receptor as well as FSH and LH receptor density in women with reduced ovarian reserve and age-related infertility. This, in turn, adversely influences granulosa cell apoptosis. Her most definitive work shows that GH co-treatment increased the receptor density for FSHR, BMPR1B, LHR, and GHR in granulosa cells compared with the non-GH-treated patients of the same age and ovarian reserve (38). Furthermore, GH restored the preovulatory down-regulation of FSHR, BMPR1B, and LHR density of the largest follicles which may consequently improve the maturation process of luteinization in older patients who have reduced ovarian reserve. The fertility of the GH-treated patients improved accordingly with a significant increase in pregnancy rate.

## Understanding Apoptosis in Peri-Ovulatory Follicles

The aforementioned studies from the PIVET-Curtin collaboration has led to a changed view regarding depletion of the ovarian reserve of primordial follicles and increased apoptosis of granulosa cells being related to poor quality oocytes in older women. On the contrary, apoptosis within the granulosa cells is an integral part of normal development and has limited predictive capability regarding oocyte quality or the ensuing pregnancy rate in IVF programs (39). In flow cytometry studies on the granulosa cells from the follicles of younger women undertaking IVF, the level of apoptosis was shown to be inversely related to the density of BMPRs as well as FSHR density. Conversely it was shown that this normal relationship became dysregulated. In the older patients the reduced apoptosis noted in the granulosa cells from the aspirated follicles at IVF (37) reflects the poor mitogenic growth turnover rate of healthy follicles rather than the death rate in an atretic follicle. It was proposed that restoring an optimum receptor density and down-regulation of receptors may improve oocyte quality (competence) with an improved pregnancy rate in older women. In fact, this has now been demonstrated in further studies on both apoptosis and the beneficial GH effects on FSH, LH, and BMP as well as GH receptors (38).

## Improving Oocyte Competence by GH for Poor Prognosis Cases in IVF

Further studies on GH adjuvant from our PIVET-Curtin collaboration have been published. One involved the detection of improved functional capacity of mitochondria in the oocytes of older women (≥35 years) treated with GH compared with an untreated group matched by age and poor-prognosis categories), as well as a young, good prognosis group (40). This study utilized immunofluorescent localization of GH receptors (GHRs) on the human oocyte and unbiased computer-based quantification of fluorescence following combined staining with mitotracker red for cellular viability, and cytochrome c oxidase for mitochondrial function. This enabled comparative assessment of oocyte quality between women of varying ages, with or without GH treatment. In this study we demonstrated for the first time, the unequivocal presence of GHRs on the human oocyte. Furthermore, the oocytes retrieved from the older women (classified as poor-prognosis) showed a significant decrease in the expression of GHRs and amount of functional mitochondria when compared with those from younger patients. Of further interest, when the older patients were treated with GH, a significant increase in functional mitochondria was observed in their oocytes. We concluded that GH exerts a direct mode of action, enabling the improvement of oocyte competence. This was achieved via the upregulation of its own receptors and enhancement of mitochondrial activity and may explain the clinical benefits from GH which we have separately reported (41, 42).

## Recent Reports Demonstrating Improved Oocyte Competence From GH Adjuvant

Accordingly, five other very recent clinical studies of GH use in IVF are of interest, beginning with a registered randomized controlled trial (RCT) from Cairo (43) where GH was added to the gonadotrophin stimulation phase of long-down regulation cycles applied in women classified as poor responders. Matching our own GH studies, the Cairo group demonstrated that significantly more usable embryos were generated under the influence of GH adjuvant. However, this did not translate into more infants, probably because of several procedural problems in their protocols as pointed out in a critical response (44), published in the same journal.

A second study from China describe the use of GH adjuvant in IVF cases categorized as RIF (45). This was an observational study where the treatment group of 22 women receiving GH injections were matched against 20 untreated cases. The GH group had both a higher pregnancy rate and live birth rate (*p* < 0.05) but this clinical aspect can easily be critically discounted on the grounds of an inadequate protocol and study design as well as low numbers. However, what was particularly interesting was the finding of elevated expression of hormone receptor (GHR) mRNA in the granulosa cells of the GH-treated group than the control group (*P* < 0.05) and the finding was positively correlated with GH levels in the follicular fluid (*r* = 0.460, *P* < 0.05). This indicated that GH adjuvant generated GHR responses which was likely to have underlined the favorable clinical responses.

A third study, again from a different province of China, compared clinical outcomes applying GH adjuvant for poor responders utilizing a mild stimulation protocol (46). The study had major design weaknesses being retrospective, the groups were not randomized, and the numbers (61 in the GH arm and 71 in the “control”) were not sufficient to determine a significant clinical improvement; requiring 200 in each arm There was however a relevant finding of significantly higher numbers of good quality “usable” embryos in the GH group (*P* < 0.01). This finding matches the study reported from Cairo (43) which was also criticized for similar reasons (44).

The most recent, fourth study, this one a prospective RCT from Iran (47), showed GH-related improvements in clinical outcomes for women classified with POR. There were 3 arms in the GnRH antagonist regimen—one (*n* = 34) utilizing GH from day 3 of the previous cycle (~20 days); a second (*n* = 32) utilizing GH from Day 8 of the gonadotrophin phase (~5 days); and a third (*n* = 28) using a GH placebo (saline injections) from Day 8 of the gonadotrophin phase. The study described significantly lower pregnancy and live birth rates from the placebo arm, and equivalent good rates from both GH adjuvant arms (20 and 5 days of GH). Whilst these favorable outcomes can be heavily discounted because of the low recruitment numbers (the GH numbers should be ~200 women and the placebo should also be ~200 women) the embryology data can be accepted as the number of collected oocytes, MII oocytes, fertilized oocytes and embryo utilization rates were all highly significantly better in the GH groups (all *P* < 0.001). This is entirely in accord with the findings reported from our center (41, 42).

A fifth study, which is now in press, examines the outcomes of GH-generated embryos which have been cryopreserved by vitrification (48). From a total 2,857 frozen embryo transfer (FET) cycles, 1,119 women had GH-generated embryos transferred. Computerized case-matching enabled 3 similar groups to be statistically analyzed for comparison, all single embryo transfers (SET) from autologous embryos—normal responders (*n* = 809) vs. poor prognosis; no GH (*n* = 201) and GH-derived embryos (*n* = 109). The pregnancy rates and live birth rates were significantly higher in the poor prognosis group where the embryos were GH-derived (*P* < 0.005 for both pregnancies and livebirths). Furthermore, tightly matched comparisons for age of the woman at FET (*n* = 89 in each group) and age of the woman at time of embryo generation (*n* = 85 in each group) showed that the GH-generated embryos had the same chance of implantation (equivalent pregnancy, live births and miscarriage rates) between the normal, good prognosis women) vs. the GH-generated poor prognosis women. This data further supports the idea that GH improves some aspect of oocyte quality which confers improved competency for implantation, and which is not detectable at morphological embryo grading.

## Adverse Clinical Effects of GH

With its known effects on growth and metabolism, it was expected that patients on GH would be at risk of sequelae such as expansion of tumors and induction of diabetes, particularly those with underlying risk factors and known insulin resistance. However, the literature on clinical GH studies does not show any serious sequelae and those which have been reported appear more related to the gonadotrophin stimulation or ovarian responses, which may sometimes reflect hyperstimulation, even in patients categorized as poor prognosis where this is not related to a low ovarian reserve. A specific reaction to GH was reported in the Jacobs study of 1995 (7) with swelling of the hands and feet along with pain in the small joints of these areas. In our decade of experience with GH we have also noted this phenomenon, albeit in only a few women (~2%) (41, 42). In each case the symptoms resolved over a few days once the GH injections were ceased. Despite the few adverse sequelae reported we would caution that case work-up requires the exclusion of tumors, particularly in the pelvis, abdominal cavity or breasts and fasting glucose undertaken to detect insipient diabetes. These aspects are part of our routine workup (49), particularly in light of the fact that ~20% of our patients will progress to utilization of GH injections. So far, most protocols in assisted reproduction utilize low-dosage (1–4 IU daily) and only for short periods ranging 10–42 days. Where longer regimens are planned, women will require review of relevant clinical features and investigation review e.g., pelvic and abdominal ultrasound scanning, mammography and serum glucose studies as indicated. Reassuringly also, the pregnancy outcomes from GH-treated women appear perfectly normal from the perspective of both the obstetric features as well as the ensuing offspring. Our findings (41, 42) are strengthened by another substantial study (50). This international collaborative study from KIMS (Kabi/Pfizer International Metabolic database) reported by Vila and her colleagues in 2015 on 201 pregnancies where women (*n* = 173) or their husbands (*n* = 28) were treated with GH for hypopituitarism. The was no relationship between GH treatment and pregnancy outcomes. None-the-less, as the over-all direction of technical, physiological and clinical studies point to the idea that unexplained and poorly explained infertility is a reflection of, so far undiagnosed, AGHD, caution must be advised. In particular, the implications of observations on Laron dwarfism, which is an autosomal recessive disorder with mutations of GHR causing insensitivity to circulating GH. Serum levels of IGF-1 are consequently low, presumably from reduced hepatic production. Apart from dwarfism, such individuals have an increased sensitivity to insulin (reducing the risk of type-2 diabetes and reduced rates of all cancers. This implies that extending GH into older people may increase the problem of insulin insensitivity (causing more diabetes) and remove the low-GH protection effect from cancers and tumors (16). Some animal researchers have proposed that longevity and good health in advanced age is traded off against reproduction, the mechanism acting via somatotrophic signaling (51).

## Conclusions

From a clinical perspective, this review article makes the case to consider that women requiring assisted reproduction and are classified as poor prognosis, may potentially be considered to have a subclinical degree of AGHD. In association with the rapidly increasing trend for delaying reproduction beyond age 35 years, GH is being widely researched now as a potential adjuvant for infertility treatment in this group who, studies consistently show, have a poorer prognosis than younger females when relying on autologous oocytes. The idea that the age-related reduction in fertility prognosis is a feature of GHD is supported by our, yet unpublished, studies showing an elevated binding protein IGFBP-3/IGF-1 ratio and this can be reduced to a normal range (matching younger, good prognosis women) by the administration of GH as an adjuvant.

In studies from different directions arising from its use as an adjuvant for IVF, it is likely that GH will be shown to have major enhancement effects on oocyte competence. Such studies should reveal major influences in the physiology of folliculogenesis and oocyte maturation. This will not only benefit older women but also younger women who currently have unexplained poor prognosis. We believe there is sufficient evidence to promote studies in two directions; firstly, to precisely define the subclinical AGHD condition among women attending fertility clinics; and secondly, to explore a more rationalized approach to the clinical use of GH. We would propose that studies should urgently be undertaken to assess whether IGF-I levels or IGFBP/IGF-I ratio can be a predictor of poor-prognosis. If so, then an RCT is required on naïve IVF cases to determine if GH adjuvant can provide a better chance for pregnancy and live birth in those predicted to have poor-prognosis.

Further studies are also required to determine appropriate and optimal dosage regimens. Currently most GH adjuvant use is applied concomitantly with the FSH-stimulation phase. However, should the GH exposure begin much earlier, at the initiation of follicle recruitment and early oocyte activation? There is also a pressing need for studies to determine if GH can favorably influence the age-related effects on aneuploidy which is a reflection of diminished oocyte competency.

## Ethics Statement

All protocols are endorsed under licence from the Reproductive Technology Council (RTC) of Western Australia (Practice Licence current to April 2021) as well as the Reproductive Technology Accreditation Committee (RTAC) under the auspices of the Fertility Society of Australia (accredited to April 2020). The reporting of any retrospective data analysis was provided by Curtin University Human Research Ethics Committee approval RD-25-10.

## Author Contributions

JY wrote the manuscript with contributions from research collaborators. SR for receptor studies. SZ for IGF-1 studies. KK for GH studies.

### Conflict of Interest

The authors declare that the research was conducted in the absence of any commercial or financial relationships that could be construed as a potential conflict of interest.
